# Inactivation of *Salmonella enterica* on post-harvest cantaloupe and lettuce by a lytic bacteriophage cocktail

**DOI:** 10.1016/j.crfs.2019.11.004

**Published:** 2019-11-28

**Authors:** Catherine W.Y. Wong, Pascal Delaquis, Lawrence Goodridge, Roger C. Lévesque, Karen Fong, Siyun Wang

**Affiliations:** a Department of Food Science, University of British Columbia, 2205 East Mall, Vancouver, BC, V6R 1Z4, Canada; b Agriculture and Agri-Food Canada, 4200 Highway 97, Summerland, BC, V0H 1Z0, Canada; c Department of Food Science and Agricultural Chemistry, McGill University, Montréal, QC, Canada; d Institute for Integrative and Systems Biology, Université Laval, Québec City, QC, Canada

**Keywords:** *Salmonella enterica*, Bacteriophage, Post-harvest, Lettuce, Cantaloupe, Biocontrol

## Abstract

*Salmonella enterica* (*S. enterica*) is a causative agent of multiple outbreaks of foodborne illness associated with fresh produce, including pre-cut melon and leafy vegetables. Current industrial antimicrobial interventions have been shown to reduce microbial populations by <90%. Consequently, bacteriophages have been suggested as an alternative to chemical sanitizers. Seven *S. enterica* strains from four serovars (10^5^ CFU/mL) were separately inoculated onto excised pieces of Romaine lettuce leaf and cantaloupe flesh treated with a five-strain bacteriophage cocktail 24 h before *S. enterica* inoculation. *S. enterica*, total aerobic populations and water activity were measured immediately after inoculation and after 1 and 2 days of incubation at 8 °C. The efficacy of the bacteriophage cocktail varied between strains. Populations of *S. enterica* Enteritidis strain S3, *S.* Javiana S203, *S.* Javiana S200 were reduced by > 3 log CFU/g and *S.* Newport S2 by 1 log CFU/g on both lettuce and cantaloupe tissues at all sampling times. In contrast, populations of strains *S.* Thompson S193 and S194 were reduced by 2 log CFU/g on day 0 on lettuce, but were not significantly different (P > 0.05) from the controls thereafter, *S.* Newport S195 populations were reduced on lettuce by 1 log CFU/g on day 0 and no reductions were found on cantaloupe tissue. Both aerobic populations and water activity were higher on cantaloupe than on lettuce. The water activity of lettuce decreased significantly (P < 0.05) from 0.845 ± 0.027 on day 0–0.494 ± 0.022 on day 1, but that of cantaloupe remained between 0.977 and 0.993 from day 0–2. The results of this study showed that bacteriophages can reduce *S. enterica* populations on lettuce and cantaloupe tissues but that the magnitude of the effect was strain-dependent.

## Highlights

• Bacteriophages reduced *S. enterica* by 1–4 log CFU/cm^2^ on lettuce and cantaloupe.
• Bacteriophage efficacy on *S. enterica* was strain-dependent on produce at 8 °C.
• *S. enterica* Enteritidis and Javiana populations were reduced by > 3 log CFU/g.
• No significant reduction of *S.* Newport S195 was observed on lettuce and cantaloupe.
• Multiplicity of infection of bacteriophage to *S. enterica* cells was 1000:1.

## Graphical abstract

**Figure:**
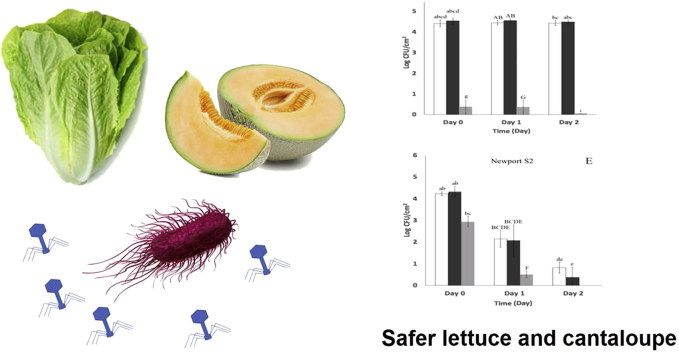

## 1 Introduction

Non-typhoidal *Salmonella enterica* (*S. enterica*) is a major etiological agent of foodborne illness (Centers for Disease Control and Prevention, 2011, Scallan et al., 2011). In the 10-year period between 1998 and 2008, leafy vegetables were the cause of 2.5% of all *S. enterica* outbreaks associated with the consumption of fresh produce in the United States (US) (Jackson et al., 2013). In 2018 alone, *S. enterica* was responsible for 16 known outbreaks in the US (Centers for Disease Control and Prevention, 2019a). Four of the 16 outbreaks (25%) were associated with plant-based foods, including frozen shredded coconut, raw sprouts, dried coconut and pre-cut melon (Centers for Disease Control and Prevention, 2019a). An outbreak of salmonellosis was definitively linked to pre-cut melon in 2019 (Centers for Disease Control and Prevention, 2019b). Recalled foods included products containing pre-cut watermelon, honeydew melon, cantaloupe and any combination of at least one of the above-mentioned fruits (Centers for Disease Control and Prevention, 2019b). Given the number of produce-related outbreaks, these incidents suggest that additional antimicrobial strategies may be required to improve the safety of fresh produce.

Leafy vegetables and many fresh-cut vegetable or fruit products are consumed without the application of a heat treatment to inactivate human pathogens. Consequently, the safety of these foods relies on the maintenance of appropriate sanitary measures during production and harvest, and application of non-thermal treatments to reduce the risk of contamination prior to distribution (Sapers, 2001). Unfortunately, non-thermal treatments such as washing in chlorinated water cannot fully eliminate enteric bacterial pathogens due to biofilm formation, attachment site inaccessibility, strength of attachment or internalization of the pathogen (Burnett et al., 2000, Carmichael et al., 1998, Costerton, 1995, Fett, 2000, Liao and Cooke, 2001, Sapers, 2001, Seo and Frank, 1999, Takeuchi et al., 2000, Takeuchi and Frank, 2000).

The use of bacteriophages as a means to inactivate bacteria was proposed soon after their discovery in 1915 (Sillankorva et al., 2012). Advantages of using bacteriophages as antimicrobial agents in foods include high specificity, self-replication, lack of negative sensory effects and general non-toxicity to humans (Moye et al., 2018, Sillankorva et al., 2012). The high specificity of bacteriophages allows for the general microbiota to remain untouched, thus avoiding undesirable effects due to alterations in spoilage patterns or the inactivation of desirable microorganisms in the food system (Sillankorva et al., 2012). Moreover, bacteriophages are tasteless and their addition does not alter the sensory characteristics or attributes of the food, which is of concern to many food processors (Moye et al., 2018).

There have been a few attempts to use bacteriophages for the control of *S. enterica* on leafy vegetables and fresh-cut melon (Leverentz et al., 2001, Spricigo et al., 2013, Zhang et al., 2019). The results of the aforementioned research have shown bacteriophages to reduce *Salmonella* populations on sprouts and lettuce (Leverentz et al., 2001, Spricigo et al., 2013, Ye et al., 2010, Zhang et al., 2019). The present work was carried out to determine the efficacy of a bacteriophage cocktail against several strains of *S. enterica* on Romaine lettuce leaf and cantaloupe tissues.

## 2 Materials and methods

### 2.1 Romaine lettuce and cantaloupe sample preparation

Whole Romaine lettuce heads and pre-cut cantaloupe were purchased from a local grocery store in Coquitlam, British Columbia, Canada. Romaine leaf sections (2 × 2 cm) (L x W) and cantaloupe flesh sections (2 × 2 × 0.2 cm) (L x W x D) were excised with a sterile sharp knife and placed in 60 mm × 15 mm Petri plates at 8 ± 1 °C (VWR International, PA, USA).

### 2.2 Bacteriophage cocktail preparation

Five *Salmonella* bacteriophages isolated from a variety of sources (Table 1) were used to formulate a cocktail. The isolates were selected on the basis of their ability to lyse at least 35 of 43 *S. enterica* strains from 31 different serotypes, including the strains used to inoculate fresh produce in the experiments described in 2.3 and 2.4 (Fong et al., 2019). Bacteriophage propagation and purification were performed according to methods described by Fong et al. (2019) and Fong et al. (2017) using *S. enterica* host strains shown in Table 1. Bacteriophage cocktail titers were measured by a plaque assay described below. Briefly, decimal dilutions were prepared in capped microcentrifuge tubes (VWR International, PA, USA) containing 450 μl Tryptic Soy Broth (TSB, Difco, Becton Dickinson, NJ, USA) + 1.0 mM CaCl_2_ (TSB-Ca). Fifty μl of *S. enterica* culture (see Table 2) prepared according to methods provided in section 2.3 were added to each tube and the contents were mixed by agitation. After 30 min incubation at 37 °C the contents were applied to the surface of Tryptic Soy Agar + 1.0 mM CaCl_2_ (TSA-Ca) in 100 mm × 15 mm petri plates. The plates were incubated at 37 °C for 24 h prior to counting the plaques. The concentration of the bacteriophage cocktail was approximately 2.5 × 10^8^ PFU/mL.

**Table 1:** Bacteriophages used in this study.

Bacteriophage	Source	Location	*Salmonella* host strain for isolation	*Salmonella* host strain for propagation
Φ3	Pretreated sludge	Montreal, QC	Enteritidis S7	Enteritidis S7
Φ6	Pretreated sludge	Montreal, QC	Javiana S1297	Javiana S1297
Felix01	Felix d’Herelle virus collection	Quebec City, QC	Typhi	Paratyphi B
HER20	Felix d’Herelle virus collection	Quebec City, QC	Newport C487-69	Newport C487-69
SE13	Sewage after first treatment	Vancouver, BC	Newport S195	Newport S195

**Table 2:** *S. enterica* strains used in this study.

Strain	Serotype	Source
S3	Enteritidis	Human
S200	Javiana	Human
S203	Javiana	Octopus
S195	Newport	Alfalfa seed
S2	Newport	Human
S193	Thompson	Spinach
S194	Thompson	Feather meal

### 2.3 Salmonella strains and inoculum preparation

The *S. enterica* strains selected for the experiments (Table 2) were previously shown to colonize lettuce seedlings (Wong et al., 2019). All were obtained from the Syst-OMICS Database (SALFOS, Université Laval, QC, Canada, https://salfos.ibis.ulaval.ca/). All strains were maintained in TSB supplemented with 20% glycerol (VWR International, PA, USA) at −80 °C. Inocula were prepared with overnight incubation at 37 °C in 10 mL TSB under agitation at 175 rpm. The overnight cultures were then spun at 1811×*g* for 10 min and the supernatant was decanted. The resulting pellets were washed twice with 10 mL 0.5 mM potassium phosphate buffer (PPB), pH 6.8–7.0 (Amresco, OH, USA). The density of the final suspension was spectrophotometrically adjusted with UV-1800 UV/Vis Spectrometer (Shimadzu, MD, USA) to an OD_600_ of 0.47–0.52 and further diluted with 0.5 mM PPB to obtain a cell density of 10^6^ CFU/mL.

### 2.4 Bacteriophage application and inoculation of Romaine lettuce and cantaloupe tissue

The bacteriophage cocktail was applied 24 h prior to inoculation with *S. enterica* to examine their ability to inactivate contaminants transferred to plant tissues during post-harvest handling. The bacteriophage cocktail (100 μL) was applied to lettuce leaf and cantaloupe flesh sections with a micro-pipettor and the liquids were gently spread over the surface using disposable sterile plate spreaders. As a control, the CaCl_2_ solution (1.0 mM) was applied to the flesh sections in parallel. The treated sections were then transferred to 60 mm × 15 mm Petri plates and stored in an incubator set at 8 ± 1 °C. A temperature of 8 °C was chosen to reflect the average expected in household refrigerators (James et al., 2017, James et al., 2008, Koutsoumanis et al., 2010). After 24 h incubation, 100 μl of each *S. enterica* inoculum was applied singly to the surface of each section in the same manner as used for the pre-treatment to achieve an inoculation level of 10^5^ CFU/mL. *Salmonella* populations were measured immediately after inoculation (day 0) and after 1 and 2 days at 8 ± 1 °C described below in 2.5.

### 2.5 Measurement of Salmonella populations on Romaine lettuce and cantaloupe tissues

Tissue sections were placed in capped test tubes with 9 mL of 0.1% (w/v) peptone (Difco, Becton Dickinson, NJ, USA) solution and agitated on a vortex mixer set on high speed for 1 min. Decimal dilutions were then prepared in 0.1% peptone and spread on Xylose Lysine Deoxycholate (XLD) Agar (Oxoid, ThermoFisher Scientific, MA, USA). Populations per surface area (CFU/cm^2^) of tissue were calculated from colony counts recorded after 24 h incubation at 37 °C.

### 2.6 Measurement of aerobic mesophilic populations on Romaine lettuce and cantaloupe tissues

Total aerobic mesophilic populations were measured on tissue sections that received no treatment prior to incubation. Dilutions prepared in 0.1% peptone as described above were applied to the surface of Tryptic Soy Agar (TSA) supplemented with yeast extract (1:200) (Oxoid, ThermoFisher Scientific, MA, USA). Aerobic populations were measured on day 0, 1 and 2 at 8 ± 1 °C. Populations per surface area (CFU/cm^2^) of tissue were calculated from colony counts recorded after 24 h incubation at 37 °C.

### 2.7 Measurement of water activity

Water activity after 0, 1 and 2 days incubation at 8 ± 1 °C was measured on tissue sections that received no treatment prior to incubation. Individual sections were placed in 35 mm × 10 mm Petri plates (Corning, NY, USA) and were warmed to 25 °C before water activity measurement with an AquaLab Series 3 water activity meter (Decagon Devices, Inc, WA, USA).

### 2.8 Statistical analyses

Five trials each were conducted on lettuce and cantaloupe tissue sections using independently grown bacterial cultures. Five sections were analyzed at each sampling time interval for each treatment: (1) *S. enterica* alone, (2) *S. enterica* + 1.0 mM CaCl_2_ and (3) *S. enterica* + bacteriophage cocktail in 1.0 mM CaCl_2_. Three to five water activity and total aerobic population measurements were obtained on day 0, 1 and 2. *S. enterica* and aerobic populations were analyzed on log_10_-transformed data by a two-way analysis of variance (ANOVA) and Tukey's honestly significant difference (HSD) for means separation. Measurements for water activity were analyzed by one-way ANOVA and Tukey's HSD for means separation. All statistical analyses were performed using RStudio, version 1.1.453 (Rstudio, Inc, MA, US).

## 3 Results and discussion

**Fig. 1:**
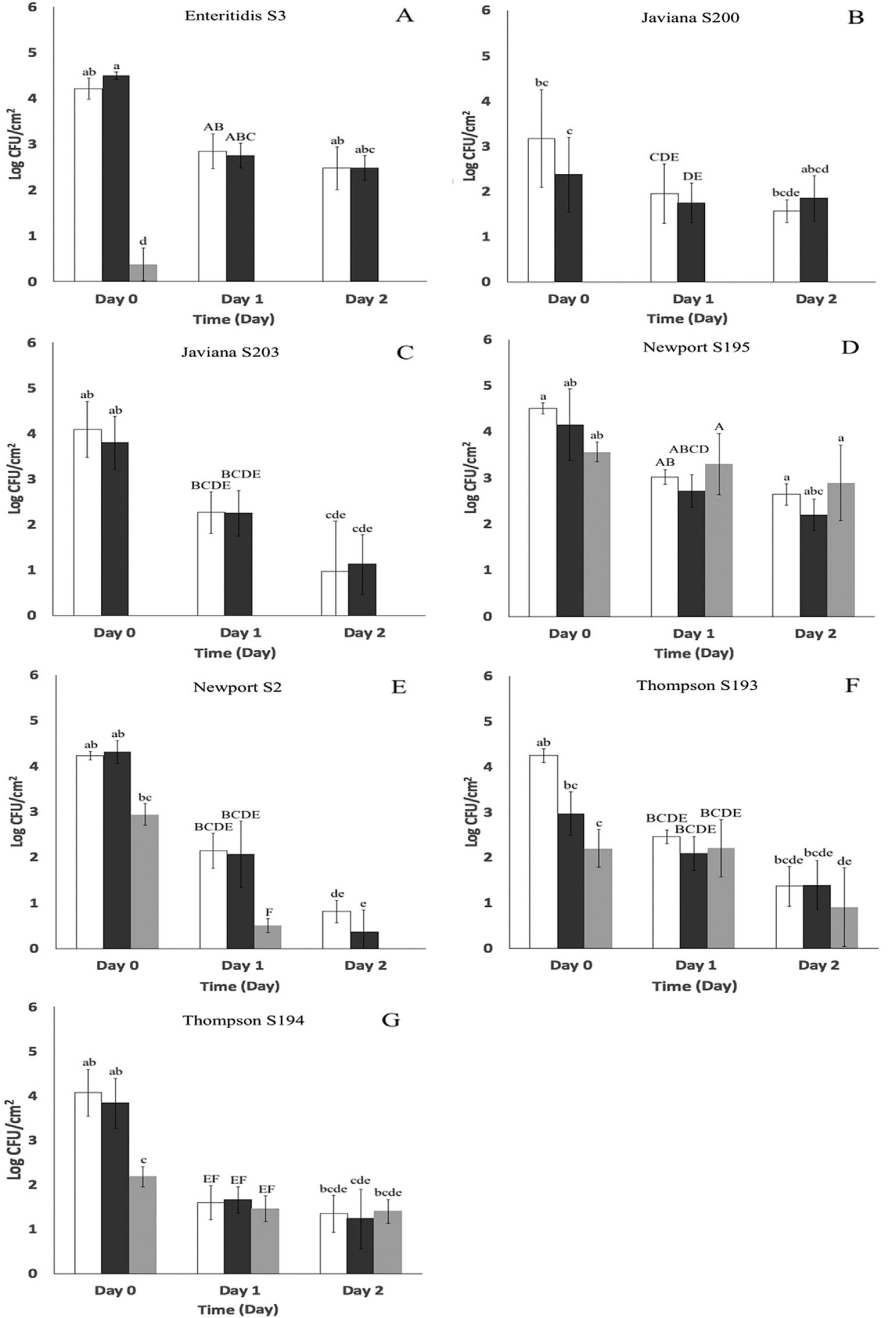
Populations (log CFU/cm^2^) of seven *S. enterica* strains on Romaine lettuce leaf tissue immediately after inoculation (Day 0) and after 1 (Day 1) and 2 days (Day 2) incubation at 8 ± 1 °C. Treatments: : untreated tissue (controls); : 1.0 mM CaCl_2_ solution applied 24 h before inoculation; : 1.0 mM CaCl_2_ + five-bacteriophage cocktail applied 24 h before inoculation. (A) *S.* Enteritidis S3. (B) *S.* Javiana S200. (C) *S.* Javiana S203. (D) *S.* Newport S195. (E) *S.* Newport S2. (F) *S.* Thompson S193. (G) *S.* Thompson S194. Different superscripts (a–d) denote significant differences (P < 0.05) between treatments on Day 0 between strains. Different superscripts (A–F) denote significant differences (P < 0.05) between treatments on Day 1 between strains. Different superscripts (a–e) denote significant differences (P < 0.05) between treatments on Day 2 between strains. Means and standard deviations were calculated using data from five biological replicates. Limit of detection is > 0.36 log CFU/cm^2^.

**Fig. 2:**
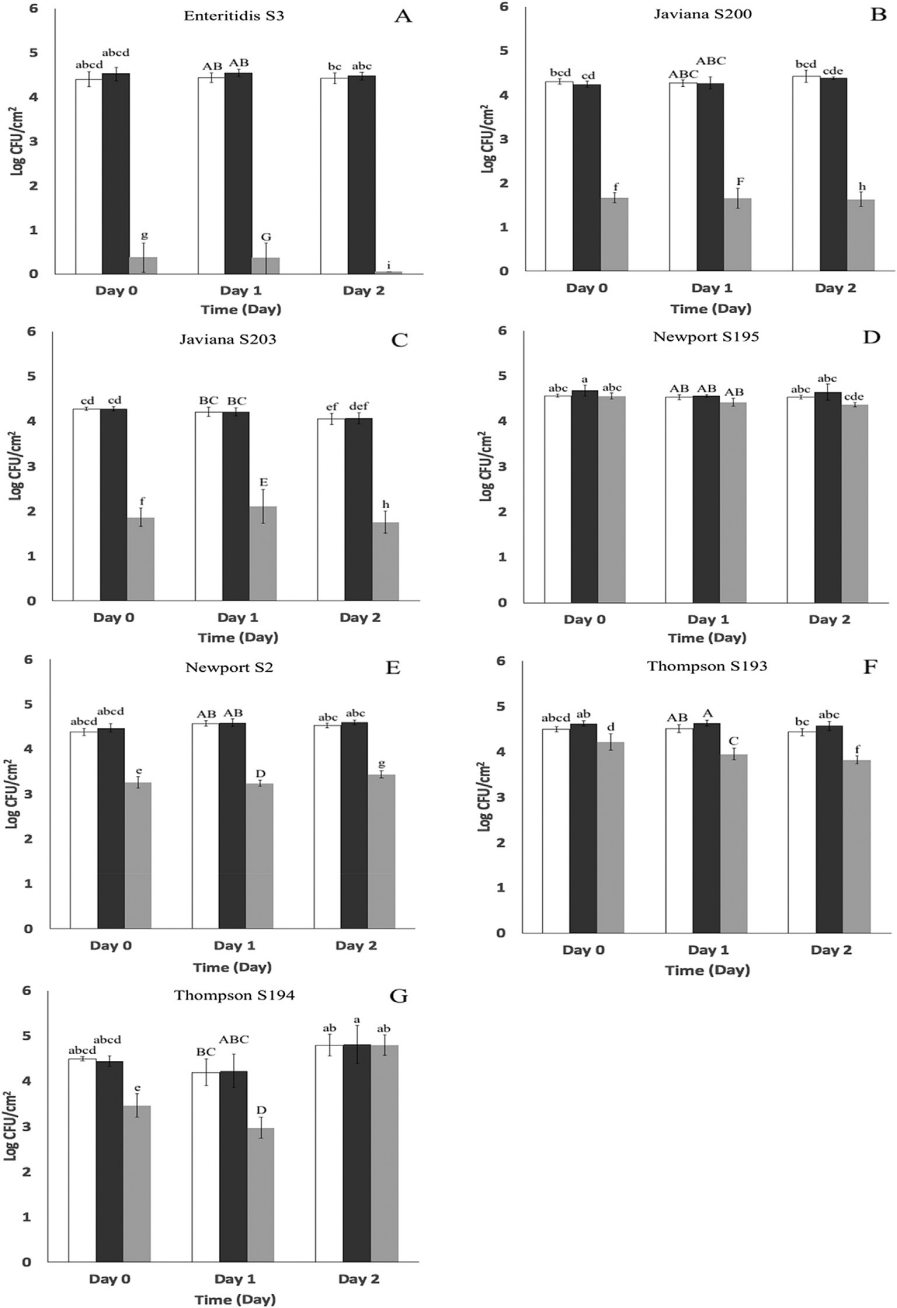
Populations (log CFU/cm^2^) of seven *S. enterica* strains on cantaloupe tissue immediately after inoculation (Day 0) and after 1 (Day 1) and 2 days (Day 2) incubation at 8 ± 1 °C. Treatments: : untreated tissue (controls); : 1.0 mM CaCl_2_ solution applied 24 h before inoculation; : 1.0 mM CaCl_2_ + five-bacteriophage cocktail applied 24 h before inoculation. (A) *S.* Enteritidis S3. (B) *S.* Javiana S200. (C) *S.* Javiana S203. (D) *S.* Newport S195. (E) *S.* Newport S2. (F) *S.* Thompson S193. (G) *S.* Thompson S194. Different superscripts (a–d) denote significant differences (P < 0.05) between treatments on Day 0 between strains. Different superscripts (A–F) denote significant differences (P < 0.05) between treatments on Day 1 between strains. Different superscripts (a–e) denote significant differences (P < 0.05) between treatments on Day 2 between strains. Means and standard deviations were calculated using data from five biological replicates. Limit of detection is > 0.36 log CFU/cm^2^.

Seven *S. enterica* strains were singly inoculated onto the surface of Romaine lettuce leaf and cantaloupe tissues treated with a cocktail consisting of equal proportions of five broad-host range lytic bacteriophage. The cocktail was applied at a density of approximately 2.5 × 10^8^ PFU/cm^2^ 24 h prior to inoculation. Fig. 1, Fig. 2 show that populations of six *S. enterica* strains (S3, S200, S203, S2, S193, S194) were significantly (P < 0.05) affected by the phage treatment. Populations of S3, S200 and S203 were reduced by up to 4 log CFU/cm^2^ and neither S200 or S203 were recovered from lettuce tissue at a level of detection = 0.36 log CFU/cm^2^ (Fig. 1, A – C). Population reductions of strains S2, S193 and S194 were lower, ranging between ~1 and 2 log CFU/cm^2^ (Fig. 1, E − G). On cantaloupe tissues, populations of strains S3, S200 and S203 were reduced by ~2–3 log CFU/cm^2^ (Fig. 2, A – C) and those of S2, S193 and S194 by ~0.5–1.5 log CFU/cm^2^ (Fig. 2, E − G). In contrast, there was no significant (P > 0.05) change in populations of strain *S.* Newport S195 measured immediately after inoculation of either lettuce or cantaloupe tissues. Differences between *S. enterica* populations on control and treated samples were sustained during subsequent incubation of lettuce tissue inoculated with strains S3, S200, S203, S2 (Fig. 1A and B, C, E), however populations of strains S193 and S194 were not significantly different (P > 0.05) after 1 or 2 days at 8 °C (Fig. 1F and G). Populations of strains S3, S200, S203, S2 and S193 also remained lower on treated cantaloupe tissue than on controls (Fig. 2A and B, C, E, F), but populations of strain S194 increased from to 2.97 ± 0.24 to 4.80 ± 0.23 log CFU/cm^2^ between day 1 and 2 (Fig. 2 G).

**Fig. 3:**
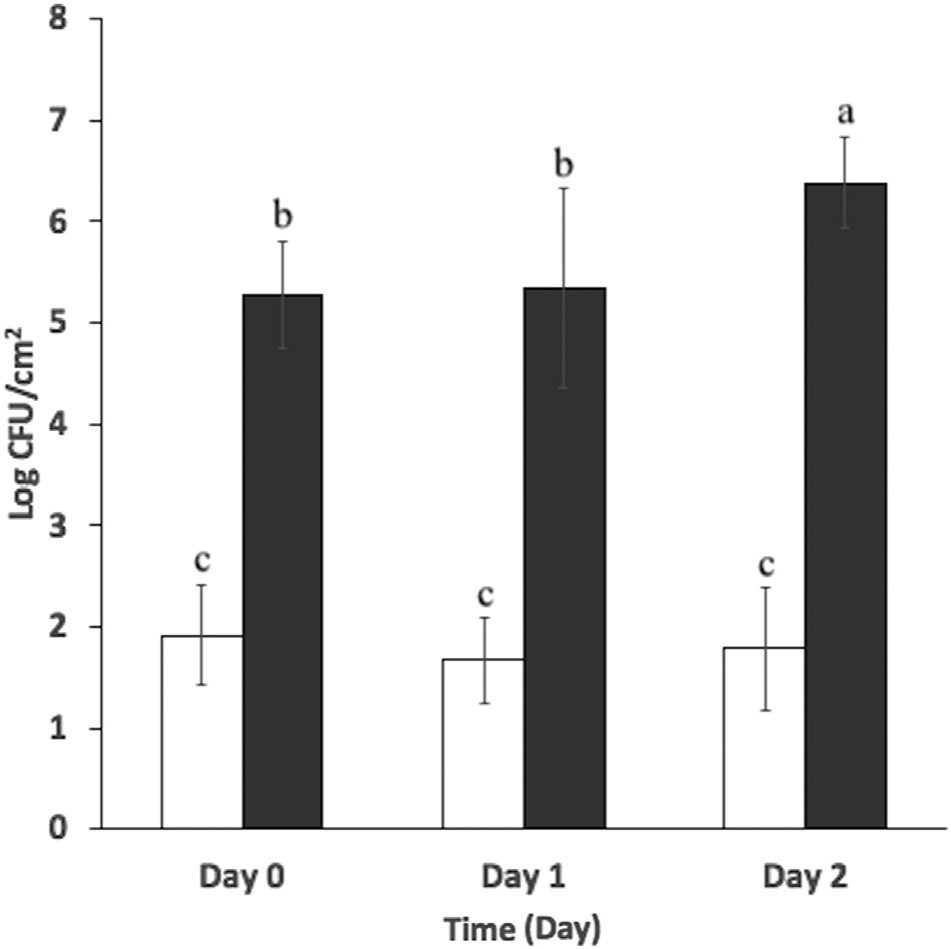
Total aerobic populations (log CFU/cm^2^) on : Romaine lettuce leaf and : cantaloupe tissues after 0, 1 and 2 days incubation at 8 ± 1 °C. Different superscripts (a–c) denote significant differences (P < 0.05) between aerobic populations on lettuce leaves and cantaloupe flesh across day 0, 1 and 2. Means and standard deviations were calculated using data from fourteen biological replicates. Limit of detection is > 0.36 log CFU/cm^2^.

The highest reductions in *S. enterica* measured in the present work occurred on day 0 against strains *S.* Enteritidis S3, *S.* Javiana S200 and S203 and *S.* Thompson S194 (Fig. 1 A – C, G). However, populations declined on lettuce after day 1 and 2 irrespective of treatment applied, likely in response to declining water activity. The water activity of lettuce and cantaloupe tissues during incubation is provided in Table 3. While measurements obtained with cantaloupe tissue remained within the range of 0.977–0.993 across all sampling times, the water activity of lettuce tissue declined significantly (P < 0.05) from 0.845 ± 0.027 to 0.494 ± 0.022 during the first 24 h of incubation but remained unchanged (0.492 ± 0.022) on day 2. An association between high water activity and total aerobic populations can be discerned. Total aerobic populations on untreated lettuce and cantaloupe tissues incubated at 8 °C for two days are provided in Fig. 3. Lettuce tissues harbored very low aerobic populations that remained between 1.67 ± 0.42 to 1.92 ± 0.50 log CFU/cm^2^ between day 0 and 2. Total aerobic populations on cantaloupe tissue were considerably higher at the outset, and increased significantly (P < 0.05) from 5.35 ± 0.98 log CFU/cm^2^ to 6.38 ± 0.45 log CFU/cm^2^ after two days of incubation at 8 °C.

**Table 3:** Water activity (a_w_)^a^ of Romaine lettuce and cantaloupe tissues after 0, 1 and 2 days of incubation at 8 ± 1 °C.

Time (days)	Romaine Lettuce^b^	Cantaloupe^c^
0	0.845 ± 0.027^a^	0.983 ± 0.002^B^
1	0.494 ± 0.022^b^	0.993 ± 0.006^A^
2	0.492 ± 0.022^b^	0.977 ± 0.002^B^

a Means and standard deviations were calculated using data from n = 3 to 5 replicates. Lettuce leaf and cantaloupe flesh sections (2 × 2 cm) were sampled at time points corresponding to day 0, 1 and 2 post inoculation.

b Different letters (a to b) in the lettuce column indicate significant differences (P < 0.05) among water activity from lettuce leaf sections on day 0, 1 and 2.

c Different letters (A to B) in the cantaloupe column indicate significant differences (P < 0.05) among water activities from cantaloupe leaf sections on day 0, 1 and 2.

A cocktail formulated from five broad-host range lytic bacteriophages applied prior to a challenge with *S. enterica* reduced the populations of several strains inoculated onto Romaine lettuce leaf and cantaloupe tissues. Inactivation of enteric bacterial pathogens on plant tissues treated with bacteriophage has been reported previously. A three-strain bacteriophage cocktail applied at a density of 7.5 × 10^7^ PFU/cm^2^ after inoculation reduced populations of *Escherichia coli* O157:H7 on cantaloupe and lettuce by 2.87 log CFU/g and 1.92 log CFU/g, respectively, after 2 days of storage at 4 °C (Sharma et al., 2009). *S. enterica* populations were reduced by 1 log CFU/g on lettuce and 0.90 log CFU/g on mung bean sprouts stored at 2 °C and 10 °C for 72 h after treatment with a commercial cocktail (SalmoFresh™) containing 10^8^ PFU/mL of six bacteriophages (Zhang et al., 2019). Leverentz et al. (2001) found that a mixture of four bacteriophage strains (SCPLX-1, Intralytix, Inc., 10^8^ PFU/mL) could reduce *S.* Enteritidis populations by 3.5 log CFU/g on honeydew melon stored at 5 °C and 10 °C. In the present work, the application of a five-phage cocktail at a density of 10^8^ PFU/cm^2^ resulted in *Salmonella* populations reductions ≥4 log CFU/cm^2^, a seemingly high degree of inactivation given that bacteriophage-based reductions of bacteria in solid foods generally range between 1 and 3 log CFU/g and that complete elimination is rare (Higgins et al., 2005, Leverentz et al., 2001, Moye et al., 2018, Whichard et al., 2003, Ye et al., 2010). However, comparisons of outcomes achieved from individual studies on bacteriophage-based control of enteric bacterial pathogens in foods is difficult due to differences in experimental design, composition of cocktails, dosage and timing of bacteriophage application and storage of target food products after treatment, among others.

While application of the bacteriophage cocktail could clearly inactivate several strains of *S. enterica*, the efficacy was both strain and plant species-dependent. Variable efficacy against different *S. enterica* strains has been reported. For example, Spricigo et al. (2013) showed that a bacteriophage cocktail could reduce *S.* Typhimurium populations by 3.9 log CFU/g on fresh-cut lettuce, but only 2.2 log CFU/g of *S.* Enteritidis. Bacteriophage cocktails for the control of foodborne bacterial pathogens are generally formulated from broad-host range bacteriophage strains to ensure activity against all potential strains of the target species (Chan et al., 2013). Despite formulation on this basis, the cocktail used in the present study was ineffective against one of the seven strains (*S.* Newport S195) on both plant tissues, an unexpected outcome as the strain was susceptible to lysis by each of the bacteriophage in the cocktail when tested by the plaque assay (Fong et al., 2017). Reasons for the lack of activity against this strain on plant tissue surfaces are unclear. One of the first initials steps in bacteriophage-host interactions is mediated by receptors on the *Salmonella* cell surface, for example vitamin B_12_ uptake outer membrane protein, flagellar or lipopolysaccharide-related O-antigen proteins (Shin et al., 2012). Expression of receptors may not occur under all environmental conditions thereby precluding binding to and subsequent infection of the bacterial cell (Bull et al., 2014), which could be the case with strain *S.* Newport S195 on lettuce or cantaloupe tissues. Moreover, mutations acquired during replication can alter host range (Drake, 1991, Oechslin, 2018, Rokyta et al., 2009). These observations reinforce the need to assess the performance of bacteriophage-based approaches to the control of enteric bacterial pathogens in fresh produce against a wide range of strains.

Overall, the cocktail examined here was more effective against *S. enterica* inoculated onto Romaine lettuce than cantaloupe tissues. Variance in the extent of control achieved in plant-based foods has been ascribed to numerous factors, including differences in chemical composition and the microtopography of surfaces colonized by target bacteria (Abuladze et al., 2008, Leverentz et al., 2004, Leverentz et al., 2001, Sharma et al., 2009). A common observation derived from studies on bacteriophage usage in foods is that the decrease in targeted bacterial populations is not consistent in different food matrices due to differences in water activity (Hudson et al., 2010, Guenther et al., 2012, Kang et al., 2013, Moye et al., 2018, Spricigo et al., 2013). The abundance of free water in moist food matrices such as beverages or sliced melons is believed to facilitate the transport of bacteriophages and to favor collision with target bacteria (Hudson et al., 2010, Moye et al., 2018). Given these assumptions, greater efficacy was anticipated against *S. enterica* on cantaloupe rather than the comparatively dry lettuce tissue surface. However, cut cantaloupe tissues can release an abundance of nutrients including readily assimilated sugars, which are present at higher concentration (0.079  g/g) than in Romaine lettuce tissues (0.011  g/g) (López et al., 2014, Perkins-Veazie et al., 2012). Microbiological analysis showed that aerobic microbial populations were several orders of magnitude greater on cantaloupe than on lettuce tissues. Hence, the presence of large background microflorae on cut cantaloupe tissues may have negatively affected the efficacy of the bacteriophage, possibly by providing other non-target attachment sites.

One of the anticipated benefits of bacteriophage-based food preservation is the ongoing release of infectious virion progeny to ensure continuous inhibition of target bacteria (Howard-Varona et al., 2017). However, a few studies have suggested that repetitive lytic bacteriophage replication cycles does not always occur under conditions used in some experimental food systems (Chibeu et al., 2013, Moye et al., 2018, Oliveira et al., 2014, Soni et al., 2012). Several factors influence host infection rates, notably temperature. In the present work, samples of inoculated plant tissues were stored at 8 °C, a temperature at the higher end of the spectrum of domestic refrigerator temperatures (James et al., 2017, James et al., 2008, Ziegler et al., 2018) but at the lower end of growth permissive temperatures for *Salmonella* spp. (D’aoust, 1993, Matches and Liston, 1968)*. S. enterica* has been reported to grow at 8 °C on leafy greens and fresh-cut cantaloupe (Huang et al., 2015, Posada-Izquierdo et al., 2015), and to survive well at 4–5 °C on leafy greens and fruits (Abd-Elall and Awadallah, 2015, Delbeke et al., 2015, Golden et al., 1993, Huang et al., 2015). Bacteriophage infection and lysis of enteric bacteria has been shown to occur at temperatures as low as 4 °C (Jurczak-Kurek et al., 2016). Inactivation of *S. enterica* at 5 and 10 °C and reports of higher activity at chill temperatures than at 20 °C provides evidence that bacteriophage retain the ability to infect *Salmonella* at low temperatures (Leverentz et al., 2001, Zhang et al., 2019).

Previous research has shown that the infectivity of bacteriophages as well as their stability during storage depends on the composition of solutions used for stabilization and preservation. Adams (1949) found that calcium ion solutions provide the highest infectivity and stability for bacteriophage T5. Other solutions were less effective for the preservation of T5, which lost its activity when stored in phosphate buffer and was inactivated when stored in citrate solution (Adams, 1949). Moreover, the infectivity of bacteriophage is known to decrease during time in storage due to interactions with solution components (Mylon et al., 2010). For example, Mylon et al. (2010) found that the MS2 bacteriophage aggregated and lost infectivity when it was stored in increasing concentrations of calcium chloride due to the neutralization of the negatively charged moieties on the surface of the bacteriophage. Given this risk, bacteriophage suspensions for use in food systems are prepared in solutions containing millimolar concentrations of calcium chloride and are used soon after preparation.

## 4 Conclusions

In summary, the results of this work have indicated that the efficacy of a bacteriophage cocktail is dependent on several factors, including the host strain and commodity. Differences in susceptibility are not limited to a specific bacteriophage but may extend to cocktails prepared from several bacteriophages. Consequently, differences in population reductions obtained with the seven strains of *S. enterica* inoculated onto lettuce leaves and cantaloupe flesh may have been a consequence of variable susceptibility to infection dictated by the host range afforded by the bacteriophage cocktail, and the concentration of the bacteriophage cocktail applied to the plant tissues.

The efficacy of the bacteriophage cocktail was strain-dependent but reduced populations by 1–4 log CFU/cm^2^ on lettuce and cantaloupe flesh sections. The cocktail was not effective against *S.* Newport S195 on both fresh produce commodities. Therefore, industrial applications may still require additional treatments or measures to further reduce *S. enterica* populations.

## Funding sources

This work was supported by grants from 10.13039/100008762Genome Canada (grant number 8505); the 10.13039/501100000038National Sciences and Engineering Research Council of Canada NSERC Discovery Grant (RGPIN-2015-04871); and the 10.13039/501100004726British Columbia Ministry of Agriculture (URACP19-211).
